# Effects of Schlemm’s Canal Suture Implantation Surgery and Pilocarpine Eye Drops on Trabecular Meshwork Pulsatile Motion

**DOI:** 10.3390/biomedicines11112932

**Published:** 2023-10-30

**Authors:** Qing Sang, Rong Du, Chen Xin, Ningli Wang

**Affiliations:** Department of Ophthalmology, Beijing Tongren Hospital Affiliated to Capital Medical University, Beijing 100730, China; sangqing@mail.ccmu.edu.cn (Q.S.); drdurong@ccmu.edu.cn (R.D.)

**Keywords:** trabecular meshwork, intraocular pressure, phase-sensitive OCT, minimally invasive glaucoma surgery

## Abstract

The trabecular meshwork is an important structure in the outflow pathway of aqueous humor, and its movement ability directly affects the resistance of aqueous humor outflow, thereby affecting the steady state of intraocular pressure (IOP). (1) Objective: The purpose of this study was to preliminarily estimate the effects of pilocarpine eye drops and trabeculotomy tunneling trabeculoplasty (3T) on trabecular meshwork (TM) pulsatile motion via phase-sensitive optical coherence tomography (Phs-OCT). (2) Method: In a prospective single-arm study, we mainly recruited patients with primary open-angle glaucoma who did not have a history of glaucoma surgery, and mainly excluded angle closure glaucoma and other diseases that may cause visual field damage. The maximum velocity (MV) and cumulative displacement (CDisp) of the TM were quantified via Phs-OCT. All subjects underwent Phs-OCT examinations before and after the use of pilocarpine eye drops. Then, all subjects received 3T surgery and examinations of IOP at baseline, 1 day, 1 week, 1 month, 3 months, and 6 months post-surgery. Phaco-OCT examinations were performed at 3 and 6 months post-surgery, and the measurements were compared and analyzed. (3) Results: The MV of TM before and after the use of pilocarpine eye drops was 21.32 ± 2.63 μm/s and 17.00 ± 2.43 μm/s. The CDisp of TM before and after the use of pilocarpine eye drops was 0.204 ± 0.034 μm and 0.184 ± 0.035 μm. After the use of pilocarpine eye drops, both the MV and CDisp significantly decreased compared to those before use (*p* < 0.001 and 0.013, respectively). The IOP decreased from baseline at 22.16 ± 5.23 mmHg to 15.85 ± 3.71 mmHg after 3 months post-surgery and from 16.33 ± 2.51 mmHg at 6 months post-surgery, showing statistically significant differences (*p* < 0.001). The use of glaucoma medication decreased from baseline at 3.63 ± 0.65 to 1.17 ± 1.75 at 3 months and 1.00 ± 1.51 at 6 months post-surgery; the differences were statistically significant (*p* < 0.001). Additionally, there was no statistically significant difference in the MV between 3 and 6 months after surgery compared to baseline (*p* = 0.404 and 0.139, respectively). Further, there was no statistically significant difference in the CDisp between 3 and 6 months after surgery compared to baseline (*p* = 0.560 and 0.576, respectively) (4) Conclusions: After the preliminary study, we found that pilocarpine eye drops can attenuate TM pulsatile motion, and that 3T surgery may reduce IOP without affecting the pulsatile motion status of the TM.

## 1. Introduction

Lowering the IOP remains the only effective treatment method for primary open-angle glaucoma (POAG), which is caused by elevated intraocular pressure (IOP) [1]. The main cause of elevated IOP is an increase in resistance to the outflow of aqueous humor. The main mechanism of the increase in aqueous humor outflow resistance in POAG is the increase in trabecular meshwork (TM) resistance and the collapse of Schlemm’s canal (SC) [2]. It was previously believed that the increase in TM resistance was mainly due to a decrease in its filtration ability. Multiple studies have identified a pulse-like outflow pattern of aqueous humor [3,4,5], which is completed via the cooperation of various tissues in the physiological pathway of aqueous outflow, including elastic TM tissues, SC, and a number of valve-like tissues, which, together, form the “pump” of aqueous outflow [6]. The decrease in the POAG outflow rate is mainly due to the abnormal increase in TM outflow resistance and SC collapse [7], and the elastic state of the TM determines its athletic ability [8]. Patients with glaucoma display abnormal TM function, where the degree of TM stiffness is 20 times higher than that of the normal population [9], mainly manifested as a decreased cell count, abnormal accumulation of the extracellular matrix, increased hardness [10,11], and compromised elastic nature of the TM/SC [12], leading to an imbalance in IOP homeostasis. At present, the influencing factors and inducing factors of TM stiffness are still poorly understood. Several factors that may affect TM stiffness include lysophospholipids, Rho kinase inhibitors, cytoskeletal-disrupting agents, dexamethasone, transforming growth factor-β2, nitric oxide, and cellular senescence [13]. Furthermore, another study found that aging is related to TM stiffness [14]. However, few studies have investigated the specific role of tissue mechanics in the progression of IOP in glaucoma [15]. TM pulsatile motion is an important part of aqueous humor efflux pumps, which can be detected via phase-sensitive optical coherence tomography (PhS-OCT), which has been validated to accurately quantify the pulsating TM motion state in the eyes [16]. PhS-OCT is one of the latest advancements that enables displacement detection with much higher sensitivity (nanometer scale) compared to the previous optical intensity-based techniques [17]. Previous studies have found that the rigidity of the TM in patients with glaucoma increases and that their pulsatile motion ability decreases [18]. The increase in the IOP in patients with glaucoma has been attributed to the increase in aqueous outflow resistance caused by the decline in TM pulsatile motion [19].

For the surgical treatment of IOP reduction, traditional surgeries mainly use non-physiological channels for filtering bleb-dependent external drainage surgery. In recent years, minimally invasive glaucoma surgery (MIGS) has become increasingly widely used in clinical practice. Among them, MIGS targeting the TM/SC uses the physiological drainage pathway of aqueous humor, in which the main mechanism of lowering the IOP is to reduce TM resistance by cutting the TM [20,21] or using a glaucoma drainage device to bypass the TM [22]. Pilocarpine nitrate eye drops are the most commonly used medication after MIGS to stimulate the contraction of the TM and pupil, thereby preventing iris adhesions [23]. However, to date, there has been no relevant research on the effects of pilocarpine eye drops and MIGS surgery on the pulsed motion of the TM.

In undertaking MIGS, our team designed a surgical technique that uses the physiological outflow pathway of aqueous humor with the TM/SC as the target, referred to as trabeculotomy tunneling trabeculoplasty (3T), also known as ab interno triple surgery. This is a type of MIGS surgery with implanted sutures in the SC. In this study, we investigated the effects of pilocarpine eye drops and triple-T surgery on TM pulsatile motion. To the best of our knowledge, this is the first study on the effects of pilocarpine eye drops and MIGS on TM pulsatile motion. PhS-OCT was used to assess changes in TM pulsatile motion before and after 3T surgery, as well as before and after the use of pilocarpine eye drops.

## 2. Materials and Methods

### 2.1. Study Design

In this prospective one-arm study, we included 22 patients (24 eyes) with primary open-angle glaucoma from the Eye Center of Beijing Tongren Hospital affiliated with Capital Medical University from March 2022 to December 2022. All subjects underwent 3T surgery, and all surgeries were performed by the same experienced ophthalmologist. All participants signed an informed consent form before participating in this study. This study followed the principles of the Declaration of Helsinki and was approved by the Ethics Committee of Beijing Tongren Hospital (Beijing, China) affiliated to Capital Medical University (MR-11-22-009185).

### 2.2. Participants

The inclusion criteria for participants were as follows: between the ages of 30 and 75 years; patients with POAG with mild-to-moderate visual field damage who meet the diagnostic criteria detailed in the China Glaucoma Guidelines (2020); no history of glaucoma surgical treatment; IOP remains uncontrolled, even with the maximum dosage of glaucoma medication; patients with rapid progress in visual field under maximum medication use; and patients who understand and voluntarily participate in the study and sign an informed consent form. The exclusion criteria for subjects were as follows: history of glaucoma surgical treatment; closed-angle glaucoma and secondary open-angle glaucoma; corneal opacity that causes an inability to visualize the angle structures; unable to cooperate in measuring the IOP due to eye conditions; factors other than glaucoma that could affect the IOP, such as orbital tumors that have caused eye compression; all possible pathologies that could give rise to visual field defect progression, like neurologic disorders, cranial trauma or neoplasms, therapy with chloroquine, etc.; and inability to sign the informed consent form.

### 2.3. Surgical Technique

All subjects underwent 3T surgical treatment. All surgeries were performed by the same experienced surgeon (NL Wang). At the beginning of the surgery, a 10–0 non-absorbable suture (Prolene, Alcon, Fort Worth, TX, USA) was tied using a surgical knot at the tip of the illuminated microcatheter (iTrack, Ellex, Menlo Park, CA, USA) after the patient received anesthesia. A 2.2 mm micro scalpel (MSL20, MANI, Utsunomiya, Japan) was used to make the main transparent corneal incision, and a viscoelastic agent (Healon GV, AMO Uppsala AB, Uppsala, Sweden) was injected into the anterior chamber to support it. A side incision was made at the 4–5 o’clock positions counterclockwise of the main incision, and a 15° bayonet (REF72-1501, Sharpiont, Reading, PA, USA) was used to make a transparent corneal auxiliary incision to enter the anterior chamber. Then, the TM was cut to approximately 2 mm, and the head end of the illuminated microcatheter was inserted into the SC and threaded counterclockwise for 360° until the microcatheter exited the TM incision. Subsequently, the connection between the suture and the microcatheter near the knot was cut, and the broken end of the suture was pulled into the anterior chamber for approximately 6 mm. Slowly, the assistant pulled back the microcatheter and injected a viscoelastic agent to expand the SC. Each clock position was injected with two clicks of a viscoelastic agent until all fiber optic microcatheters had exited the SC. A lamellar scleral tunnel was created on the surface of the nasal sclera and a bayonet was pierced under the scleral tunnel, before entering it into the anterior chamber. Two sections of stitching were hooked out and tied together. The surgery was concluded after cleaning the anterior chamber.

### 2.4. Assessments

All subjects underwent baseline examinations, including IOP, slit lamp, gonioscopy, optical coherence tomography (OCT) of the optic nerve head, PhS-OCT, visual field, anterior chamber depth, corneal endothelium, and axial examination. All subjects underwent two PhS-OCT examinations prior to surgery, one before and one after the use of 2% pilocarpine eye drops. Pilocarpine eye drops were administered every 15 min, continually for four applications, before the PhS-OCT examination was performed. IOP and records of glaucoma medication use were obtained during follow-up visits at post-operative day 1, week 1, month 3, and month 6. PhS-OCT examinations were performed at 3 and 6 months post-surgery.

### 2.5. IOP Examinations

A applanation tonometer (AT020, Carl Zeiss, Visucam Meditec, Germany) was used to measure the IOP during each follow-up, with an average of three measurements taken. The examination was conducted by the same experienced examiner, with the subject sitting in the examination position. The examination time was uniformly selected between 9:00 a.m. and 11:00 a.m. during each follow-up.

### 2.6. PhS-OCT Examinations

**Figure 1:**
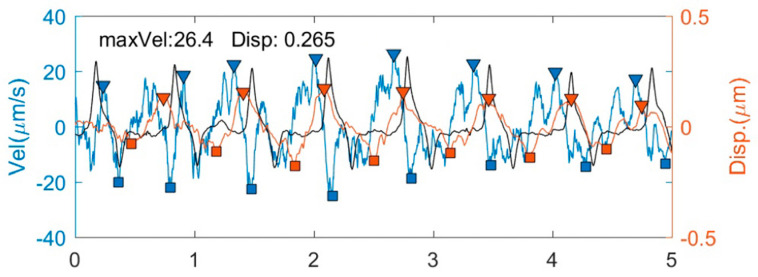
Parameters on trabecular meshwork motion captured and calculated via phase−sensitive optical coherent tomography. The black trajectory represents the heartbeat; the blue trajectory represents the instantaneous velocity (Vel) of the trabecular meshwork during one cardiac cycle; and the orange trajectory represents the cumulative displacement (Disp) of the trabecular meshwork motion. The maximum velocity (maxVel) refers to the highest velocity during one cardiac cycle.

Regarding the maximum velocity (MV) and cumulative displacement (CDisp) of the TM (Figure 1), PHS-OCT (laboratory prototype) was used to measure the MV and CDisp of the TM pulsatile motion. PhS-OCT can perform dynamic elastography of the TM [24]. It was possible for the external region of the TM to be affected by the sutures, given that a 2–3 mm incision was made on the nasal side of the TM during the surgical procedure, with sutures implanted within the SC. Therefore, we chose to perform the examination within the nasal TM region (3 or 9 o’clock position) of the surgical eye. All examinations were conducted in the same room under consistent lighting conditions. Each eye was scanned three times using the same method, and the average of the three scans was taken as the result. The examination was conducted by the same experienced examiner, with the subject sitting in the examination position. The examination time was uniformly selected between 9:00 a.m. and 11:00 a.m. during each follow-up. The clinical PhS-OCT prototype consisted of three components: a spectral domain OCT system; a digital pulsimeter; and an external control unit, which was used to synchronize OCT data acquisition and cardiac signal recording [25]. The theoretical axial resolution was approximately 5.5 µm, and the lateral resolution was approximately 16 µm. For each imaging session, 2000 OCT B-scans were acquired at a rate of 400 B-scans per second, with a duration of 5 s. In the transient regime, propagating shear waves were induced in the sample, and their propagation was tracked with PhS-OCT. The stiffness of the sample was then characterized by retrieving the shear modulus from the shear wave propagation speed [26]. In order to present the propagation of mechanical waves within a single B-scan cross section and calculate the displacement between successive B-scans, phase information was extracted [27]. Between adjacent B-scans, we analyzed the phase shift of each pixel in the OCT signals and then calculated the instantaneous velocity based on the difference between the two B-scan images, as described in our previous study [28]. One-third of the distance anterior to the sclera spur along the line between Schwalbe’s line and the sclera spur was defined as the internal region of the TM. The MV was defined as the maximum value on the velocity waveform, while the CDisp was defined as the integration of the velocity waveform within one cardiac cycle (Figure 1) [25]. For detailed principles and methods on how PhS-OCT conducts CDisp and MV measurements, please refer to our previous studies [16].

### 2.7. Data Analysis

SPSS 26.0 (IBM, Chicago, IL, USA) software was employed to conduct statistical analyses of the data. Measurement data, such as the IOP, TM movement speed, and TM movement amplitude, were tested for normality. The continuous variables conforming to the normal distribution were described by ±s, and the comparison of subjects before and after surgery was performed using the paired sample *t*-test; one-way analysis of variance (ANOVA) was used for multiple group mean comparisons; if the data did not conform to the normal distribution, they were described as medians and 25–75%, and underwent non-parametric statistical tests. The difference was statistically significant at *p* < 0.05.

## 3. Results

### 3.1. Demographics and Baseline Characteristics

This study included twenty-two patients with POAG who required surgical treatment, two of whom underwent binocular surgery. Thus, a total of 24 eyes were included in this study. All subjects completed a follow-up 6 months after surgery. All subjects were diagnosed with POAG and had no history of undergoing glaucoma surgery. The average age of all subjects was 43.29 ± 10.73 years, and 90% of the subjects had used ≥4 IOP-lowering drugs before surgery. The average use of glaucoma medication was 3.63 ± 0.65, and the average IOP under maximum pre-operative medication was 22.16 ± 5.23. The baseline examinations and the results of the subjects are shown in Table 1.

### 3.2. IOP and Medications

**Figure 2:**
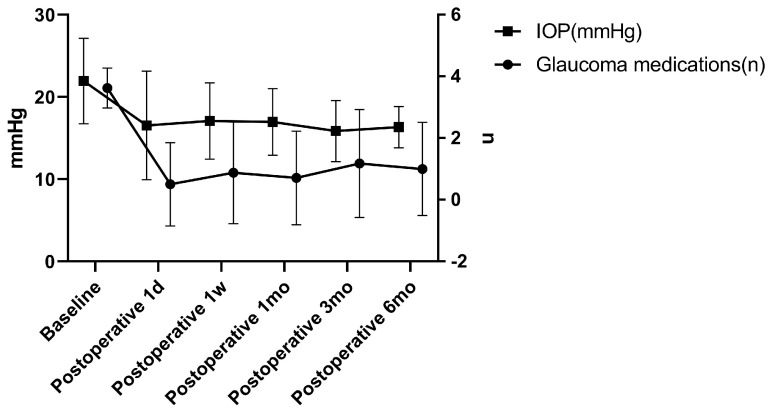
Intraocular pressure and glaucoma medications. IOP: intraocular pressure; d: day; w: week; and mo: month.

A total of 22 participants with 24 eyes were included in this study without grouping. At 3 months and 6 months post-surgery, one eye from each patient underwent a secondary trabeculectomy due to elevated intraocular pressure, and thus they were not included in the statistics. Data from 24 eyes were analyzed at 1 day, 1 week, and 1 month; data from 23 eyes were analyzed at 3 months; and data from 22 eyes were analyzed at 6 months post-surgery. After surgery, 52.9% of the patients’ IOP decreased by more than 30%, and 70.6% of the patients’ glaucoma medication use decreased by ≥2 types. At 6 months post-surgery, 64.7% of the participants no longer needed to continue using glaucoma medication. Table 2 and Figure 2 present the pre- and post-operative IOP and glaucoma medication usage of the participants. The IOP and glaucoma medication use of the participants at 1 day, 1 week, 1 month, 3 months, and 6 months post-surgery were both significantly lower than those pre-surgery (*p* < 0.05). The IOP decreased from baseline at 22.16 ± 5.23 mmHg to 16.55 ± 6.60 mmHg at 1 day, 17.08 ± 4.63 mmHg at 1 week, 16.97 ± 4.05 mmHg at 1 month, 15.85 ± 3.71 mmHg at 3 months, and 16.33 ± 2.51 mmHg at 6 months post-surgery. The use of glaucoma medication decreased from baseline at 3.63 ± 0.659 to 0.50 ± 1.35 at 1 day, 0.88 ± 1.65 at 1 week, 0.71 ± 1.51 at 1 month, 1.17 ± 1.75 at 3 months, and 1.00 ± 1.51 at 6 months post-surgery (Table 2 and Figure 2).

### 3.3. The MV and CDisp of the TM after Surgery

**Figure 3:**
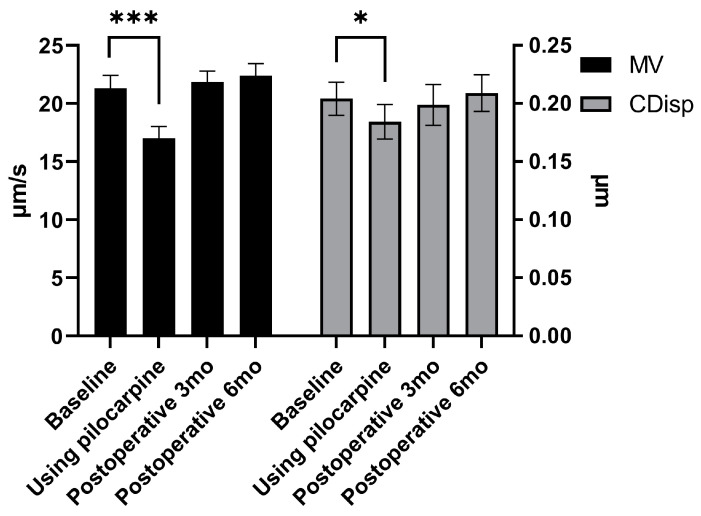
The MV and CDisp of the TM before and after the use of pilocarpine eye drops, as well as before and after the surgery. MV: maximum velocity, and CDisp: cumulative displacement. mo: month. After the use of pilocarpine eye drops, both the MV and CDisp significantly decreased compared to before use (*p* < 0.001 and 0.013, respectively). * and ***: the difference is statistically significant.

The pre-operative and post-operative pulsatile TM motion results of the subjects are shown in Table 3 and Figure 3. The MV before surgery, 3 months post-surgery, and 6 months post-surgery was as follows: 21.32 ± 2.63 μm/s, 21.85 ± 2.28 μm/s, and 22.38 ± 2.38 μm/s, respectively. The CDisp before surgery, 3 months post-surgery, and 6 months post-surgery was as follows: 0.204 ± 0.034 μm, 0.199 ± 0.041 μm, and 0.209 ± 0.037 μm, respectively. There was no statistically significant difference in the MV between 3 and 6 months after surgery compared to baseline (*p* = 0.404 and 0.139, respectively). Furthermore, there was no statistically significant difference in the CDisp between 3 and 6 months after surgery compared to baseline (*p* = 0.560 and 0.576, respectively) (Table 3 and Figure 3).

### 3.4. The MV and CDisp of the TM before and after Pilocarpine Eye Drops

Two phase-OCT examinations were conducted on all subjects, one before and one after using the pilocarpine eye drops. The MV and CDisp before and after the use of pilocarpine eye drops were 21.32 ± 2.63 μm/s and 17.00 ± 2.43 μm/s, and 0.204 ± 0.034 μm and 0.184 ± 0.035 μm, respectively. After the use of pilocarpine eye drops, both the MV and CDisp significantly decreased compared to before use (*p* < 0.001 and 0.013, respectively) (Table 4 and Figure 3).

## 4. Discussion

In this study, we preliminarily investigated the effects of pilocarpine eye drops and MIGS on TM pulsatile motion. To the best of our knowledge, this is the first study on the effects of pilocarpine eye drops and MIGS on TM pulsatile motion.

**Figure 4:**
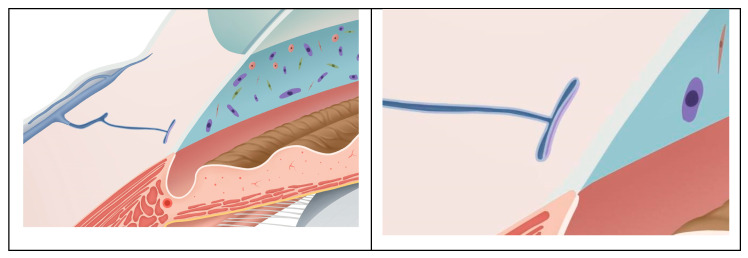
A simulated representation of the Schlemm’s canal morphology before the surgery. The left image shows a schematic diagram of the anterior chamber angle structure before the surgery, where the Schlemm’s canal is in a collapsed state. The right image is an enlarged schematic diagram of the Schlemm’s canal, and the purple area represents the variation in the cross-sectional area of the Schlemm’s canal caused via the pulsatile movement of the trabecular meshwork.

**Figure 5:**
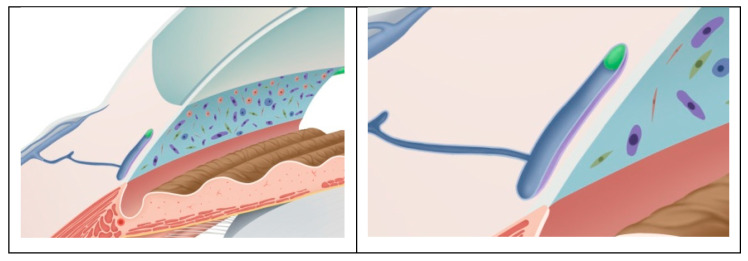
A simulated representation of the Schlemm’s canal after the surgery with suture implantation. The left image shows a schematic diagram of the anterior chamber angle structure after the surgery, where the Schlemm’s canal is in an expanded state. The right image is an enlarged schematic diagram of the Schlemm’s canal, and the purple area represents the variation in the cross-sectional area of the Schlemm’s canal caused via the pulsatile movement of the trabecular meshwork.

The post-operative IOP and the use of glaucoma medications were both lower than before surgery in these subjects. After 3T surgery, 52.9% of the patients’ IOP decreased by more than 30%, and 70.6% of the patients’ glaucoma medication use decreased by more than or equal to two types of medications. In addition, 64.7% of the participants no longer needed to continue using their glaucoma medication. For the OAG patients, if the visual field continues to progress after treatment with intraocular pressure-lowering drugs, 3T surgical treatment may be performed to avoid the progression of this condition. More suitable for patients with a normal-angle structure, for patients with a history of trabeculectomy surgery, changes in the angle structure of the atrium may ultimately result in the inability to perform 3T surgery due to previous surgeries. Therefore, we suggest that it is a better indication for patients who do not have a history of glaucoma surgery treatment. Trabeculotomy tunneling trabeculoplasty’s success can be attributed to its mechanism of lowering the IOP, which leads to favorable outcomes. The 3T surgical procedure involved partial incisions of the TM to reduce TM resistance while simultaneously using viscoelastic agents and internal sutures to expand the SC, thereby reducing aqueous humor outflow resistance. The SC can be stretched and prevented from collapsing using the tension sutures placed within it for an extended period, as shown in Figure 4 and Figure 5.

**Figure 6:**
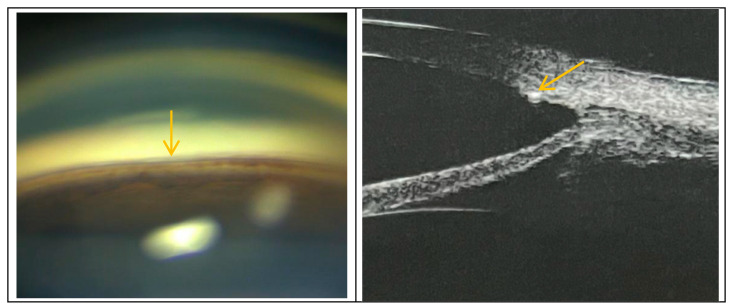
The left image shows the post-operative gonioscopy examination of the subjects, and the right image shows the post-operative ultrasound biomicroscopic examination of the subjects. The position indicated by the yellow arrow in the figure represents the 10–0 suture implanted inside the Schlemm’s canal on the side where the surgical forceps were used during the operation. The suture is located near the upper wall of the Schlemm’s canal.

Our results revealed no statistically significant differences in the MV and CDisp after 3T surgery compared to baseline, suggesting that 3T surgery exerts no clear effect on the pulsatile movement of the TM. Although the inner wall of the SC was pulled in after the suture was placed, it did not have a significant impact on its pulsatile movement. We believed that the location of the suture inside the SC could be the cause. The suture was found to be positioned near the upper wall of the SC, as shown in Figure 5 and Figure 6, based on post-operative gonioscopy and ultrasound biomicroscopic examination.

Following the use of pilocarpine, the MV and CDisp were lower than those without pilocarpine, which suggests that pilocarpine eye drops can reduce TM pulsatile motion. Pilocarpine stimulates cholinergic activity in the TM cells, leading to contraction [23]. This may also function to stimulate the contraction of the iris sphincter muscle to reduce the size of the pupil [29]. In this scenario, the pupils cannot dilate and contract freely, and the movement of the iris is restricted, closely related to the TM and SC. The restricted movement of the iris may lead to the limited movement of the TM. Dissecting the ciliary muscle from the scleral spur/trabecular meshwork completely abolishes the effect of pilocarpine [23]. Thus, the effect of pilocarpine on influence outflow facility is solely mediated via the contraction of the ciliary muscle, which affects the geometry of the TM and SC, with no direct drug effect on the TM/SC [30]. The ciliary muscle and the choroid form an elastic network in terms of function, extending from the TM all the way to the back of the eyes [31]. The ciliary muscle controls the accommodative movement of this elastic network; by relaxing the posterior attachments of the ciliary muscle, not only can its accommodative amplitude be enhanced, but it may also contribute to improving the flow of the aqueous humor through the trabecular meshwork [32]. In contrast, when the ciliary muscle contracts, it may weaken the amplitude of motion in this elastic network. The TM is a component of this elastic network; therefore, we speculated that pilocarpine’s constriction effect on the ciliary muscle attenuates the pulsatile motion of the TM.

In this study, Phs-OCT was used to detect the MV and CDisp, which have been proven to be highly accurate and repeatable in TM pulse motion detection [6,16,33]. PhS-OCT may prove to be an effective tool for the evaluation of functional properties of the outflow system [34]. Additionally, there is evidence to suggest that randomly measured IOP data in outpatient clinics do not reflect the true level of the IOP [35,36]. Indeed, for patients with open-angle glaucoma, even if the IOP is controlled, the visual field may still progress; thus, it is crucial to explore the pattern of aqueous humor discharge and IOP homeostasis using a method that can detect the long-term state of the IOP, similar to the relationship between random blood glucose measurements and glycated hemoglobin measurements in patients with diabetes. Therefore, using PhS-OCT to examine TM pulse motion provides a method for observing long-term IOP stability.

**Figure 7:**
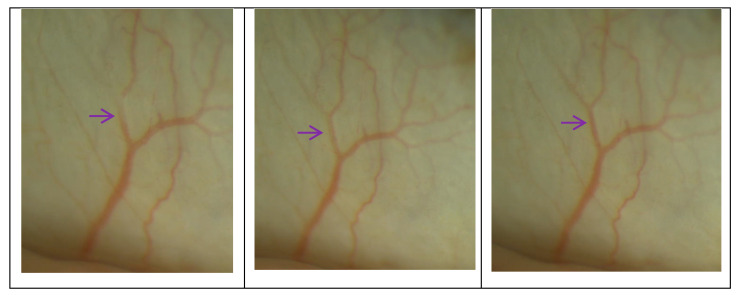
Aqueous veins captured using a slit lamp camera. The purple arrows in the image indicate the dynamic changes in aqueous humor in the Schlemm’s canal after surgery, and the outflow of aqueous humor within it still exhibited pulsatile flow.

After 3T surgery, the SC was expanded. Due to the unchanged MV and CDisp of the TM, this expansion also increased the average change in the SC cross-sectional area due to the motion of the TM. As shown in Figure 5, the cyclic volume changes of the SC increased, which may have implications for improving aqueous outflow and IOP regulation. Post-operative volume variability increased due to the SC serving as a reservoir for aqueous humor. The physiological outflow pattern of aqueous humor has been confirmed as a pump regulation theory, the mechanism of which involves changes in the IOP, causing compliant deformation of the TM, which leads to a cascading movement of valve-like tissues within the SC [19]. Previous studies in live subjects [36] have observed a pulsatile outflow of aqueous humor [37]. Using slit lamp photography examinations of patients after 3T surgery, we also observed that the outflow pattern of the aqueous humor still exhibited a pulsatile flow. As shown in Figure 7, the inner walls of the TM and SC were closely connected and moved together; therefore, the movement of the TM determines the periodic changes in the SC volume, whereby the greater the periodic change in the SC volume caused via TM movement, the more aqueous humor is discharged. Thus, the SC serves as a reservoir for aqueous humor, and its post-operative volume variability increases.

In previous research on viscocanalostomy (canaloplasty) surgery, it was considered that the post-operative enlargement of the SC is an important mechanism for reducing the resistance of aqueous humor outflow [38,39]. Our results suggests that it may be necessary to consider the changes in the TM pulsatile motion. The changes in the motion state of the TM were as equally important as the changes in the SC diameter, and the motion state of the TM directly affected the level of outflow resistance of the aqueous humor.

When measuring the pulsatile movement of the TM, the area of lateral measurement was defined as the external movement area of the TM, which is different from that used in other studies. The TM was tensioned, to a certain extent, after the patients in this study were loaded with tension sutures in the SC after surgery, and its overall width was reduced, which affected the accuracy of internal movement measurements. Therefore, the external area of the TM was selected as the target area for this study.

The limitations of this study include the failure to obtain pre-operative data on the SC diameter for the participants in this group. The cohort of patients was rather small and the follow-up period was relatively short. Additionally, the investigation of the TM pulsatile motion may have been influenced by different IOPs, ages, and physical conditions, leading to varied results. In the future, our research team will expand the sample size and extend the observation period. We will aim to conduct more in-depth research on the pulsatile motion of the trabecular meshwork with respect to different ages, IOP levels, and physical conditions.

## 5. Conclusions

Following the preliminary study, our results revealed that in patients with POAG, the IOP successfully decreased after 3T surgery, which was accompanied by an increase in the periodic changes in the SC volume, resulting in increased aqueous humor drainage. This suggests that the post-operative pump function for aqueous humor outflow may have been enhanced. For the first time, we discovered that pilocarpine, as a common drug for glaucoma, can reduce the pulsed movement of the trabecular meshwork, but its mechanism requires further investigation. Quantitative detection of pulsatile TM motion via PhS-OCT provided important support for studying the pump function status of aqueous humor outflow and may be applied in clinical practice in the future. The results of this study contribute to our understanding of changes in aqueous humor outflow patterns after surgery. As one type of MIGS surgery, 3T surgery was clinically researched for the first time to reveal the effect of suture implantation in the SC on TM pulsatile motion and aqueous humor outflow pump function, offering new directions and considerations for clinical research and treatment.

## Figures and Tables

**Table 1:** Demographic of subjects.

	All Participants
Age (years)	43.29 ± 10.73
Sex (F:M)	7/15
Eyes (n)	24
POAG	
Mild (n)	4
Moderate (n)	17
Severe (n)	3
Central corneal thickness (μm)	544.96 ± 47.84
Axial length (mm)	24.57 ± 1.50
Number of corneal endothelial cells (mm^2^)	2584.8 ± 290.53
Intraocular pressure (mmHg)	22.16 ± 5.23
Mean deviation (dB)	9.63 ± 3.22
Anterior chamber depth (mm)	3.08 ± 0.33
Glaucoma medications (n)	3.63 ± 0.65
Heart rate (times/minute)	72.25 ± 6.07
Mean arterial pressure (mmHg)	122.04 ± 15.08

POAG: primary open-angle glaucoma, F: female, and M: male.

**Table 2:** Intraocular pressure and glaucoma medications.

	Baseline (n) Mean ± SD	Post-Surgery (Time) (n)	Mean ± SD	Mean Difference	*p*-Value
Intraocular pressure (mmHg)	22.16 ± 5.23 (24)	1 day (24)	16.55 ± 6.60	5.61 ± 7.16	0.002
		1 week (24)	17.08 ± 4.63	4.83 ± 7.35	0.005
		1 month (24)	16.97 ± 4.05	6.30 ± 6.14	0.001
		3 months (23)	15.85 ± 3.71	6.61 ± 4.78	<0.001
		6 months (22)	16.33 ± 2.51	6.03 ± 4.78	<0.001
Glaucoma medications (n)	3.63 ± 0.65 (24)	1 day (24)	0.50 ± 1.35	3.13 ± 1.36	<0.001
		1 week (24)	0.88 ± 1.65	2.75 ± 1.57	<0.001
		1 month (24)	0.71 ± 1.51	2.92 ± 1.47	<0.001
		3 months (23)	1.17 ± 1.75	2.44 ± 1.59	<0.001
		6 months (22)	1.00 ± 1.51	2.59 ± 1.37	<0.001

**Table 3:** Pulsatile TM motion before and after surgery.

	Baseline (n) Mean ± SD	Post-Surgery (Months) (n)	Mean ± SD	Mean Difference	*p*-Value
MV (μm/s)	21.32 ± 2.63 (24)	3 months (24)	21.85 ± 2.28	−0.520 ± 3.004	0.404
		6 months (22)	22.38 ± 2.38	−1.050 ± 3.360	0.139
CDisp (μm)	0.204 ± 0.034 (24)	3 months (24)	0.199 ± 0.041	0.005 ± 0.043	0.560
		6 months (22)	0.209 ± 0.037	−0.004 ± 0.042	0.576

MV: maximum velocity, and CDisp: cumulative displacement.

**Table 4:** The MV and CDisp of the TM before and after the use of pilocarpine eye drops.

	Eyes, n	Baseline Mean ± SD	After Pilocarpine	Mean Difference	*p*-Value
MV (μm/s)	24	21.32 ± 2.63	17.00 ± 2.43	4.32 ± 2.68	<0.001
CDisp (μm)	24	0.204 ± 0.034	0.184 ± 0.035	0.020 ± 0.036	0.013

MV: maximum velocity, and CDisp: cumulative displacement.

## Data Availability

The datasets used and analyzed during the current study are available from the corresponding author upon reasonable request.
